# Tuberculous Meningitis: Pathogenesis, Immune Responses, Diagnostic Challenges, and the Potential of Biomarker-Based Approaches

**DOI:** 10.1128/JCM.01771-20

**Published:** 2021-02-18

**Authors:** Charles M. Manyelo, Regan S. Solomons, Gerhard Walzl, Novel N. Chegou

**Affiliations:** a DST/NRF Centre of Excellence for Biomedical Tuberculosis Research, South African Medical Research Council Centre for Tuberculosis Research, Division of Molecular Biology and Human Genetics, Faculty of Medicine and Health Sciences, Stellenbosch University, Cape Town, South Africa; b Department of Paediatrics and Child Health, Faculty of Medicine and Health Sciences, Stellenbosch University, Cape Town, South Africa; Emory University

**Keywords:** biomarker, central nervous system infections, diagnosis, immune response, meningitis, pathogenesis, tuberculosis, tuberculous meningitis

## Abstract

Tuberculous meningitis (TBM) is the most devastating form of tuberculosis (TB), causing high mortality or disability. Clinical management of the disease is challenging due to limitations of the existing diagnostic approaches. Our knowledge on the immunology and pathogenesis of the disease is currently limited. More research is urgently needed to enhance our understanding of the immunopathogenesis of the disease and guide us toward the identification of targets that may be useful for vaccines or host-directed therapeutics. In this review, we summarize the current knowledge about the immunology and pathogenesis of TBM and summarize the literature on existing and new, especially biomarker-based, approaches that may be useful in the management of TBM. We identify research gaps and provide directions for research which may lead to the development of new tools for the control of the disease in the near future.

## Abstract (precis)

Tuberculous meningitis (TBM) is the most devastating form of tuberculosis (TB), causing high mortality or disability. Clinical management of the disease is challenging due to limitations of the existing diagnostic approaches. Our knowledge on the immunology and pathogenesis of the disease is currently limited. More research is urgently needed to enhance our understanding of the immunopathogenesis of the disease and guide us toward the identification of targets that may be useful for vaccines or host-directed therapeutics.

## INTRODUCTION

Tuberculosis (TB) is the leading cause of death from a single infectious agent (Mycobacterium tuberculosis) and killed nearly 1.5 million people in 2018 (1). TB mostly manifests as a pulmonary disease but also affects other body sites, causing extrapulmonary TB (EPTB). About 5% of all EPTB cases are tuberculous meningitis (TBM), which results from the spread of M. tuberculosis into the meninges and cerebrospinal fluid (CSF) (2). It is unclear what proportion of all TB cases are TBM, as it varies across studies by local TB prevalence, with high proportions (about 10%) suggested in high TB burden settings compared to low TB prevalence settings (around 1%) (3). It is estimated that at least 100,000 individuals develop TBM annually (3). TBM is the most devastating form of TB and continues to cause high morbidity and mortality (4), with an estimated 50% of patients dying or suffering neurological sequelae and complications (5, 6). TBM is mostly common in young children (2 to 4 years old) and individuals infected with HIV (4, 7). Besides TBM, infectious meningitis is also commonly caused by viruses, bacteria, and fungi, which are often challenging to differentiate from meningitis caused by TB (8). In both children and adults, viral meningitis is more common, followed by bacterial and fungal meningitis (9–11). Streptococcus pneumoniae is the most common cause of bacterial meningitis worldwide in both adults and children, followed by Neisseria meningitidis (8, 12). TB (22%) was reported as the most common form of bacterial meningitis in children from a high TB burden setting, followed by Streptococcus pneumoniae (4%) and Klebsiella pneumoniae (3%) (11). The diagnosis of TBM is challenging and often delayed, with deleterious outcomes for patients. These challenges are even more serious in very young infants (13).

The methods currently used for diagnosing TBM in children are unreliable. Symptoms and signs of the disease are not specific, and the tests used for diagnosis of the disease are highly invasive and time-consuming. General diagnostic tests, including CSF white cell count (WBC) with differential, total protein, and glucose level measurements, are performed for the diagnosis of meningitis (14). Typical CSF findings in TBM include increased total protein, decreased CSF-to-serum glucose ratio, and increased total WBC with lymphocytic pleocytosis (14, 15). Bacterial meningitis is characterized by mild-to-marked elevated total protein, mild-to-marked decreased CSF-to-serum glucose ratio, and increased total WBC with neutrophil predominance (16). In viral meningitis, there are normal-to-elevated levels of total protein, usually normal CSF-to-serum glucose ratio, and minimal total WBC, with lymphocyte predominance; while fungal meningitis is characterized by elevated total protein, low CSF-to-serum glucose ratio, and minimal total WBC with lymphocyte predominance (14). Various diagnostic algorithms that take into account the symptoms and signs, in conjunction with results from laboratory and imaging tests, have been suggested for use in classifying individuals suspected of having TBM, at least for research purposes (17, 18). The clinical management of TBM is challenging due to an incomplete understanding of the immunopathogenesis underlying the disease. Further investigations are required to update and refresh the body of knowledge for management of the disease, including the development of effective TB drugs, host-directed therapies, vaccines, and diagnostics. In the current review, we summarize evidence published in the literature on different diagnostic approaches for TBM in children, the pathogenesis and immunology of TBM, and the recent advances in the search for novel approaches, mainly biomarkers, in the diagnosis of the disease.

## IMMUNOPATHOGENESIS OF TBM

### Pathogenesis.

The development of TBM begins with respiratory infection, followed by hematogenous spread to the central nervous system (CNS). Within the lungs, a localized infection is initiated following inhalation of aerosol droplets containing M. tuberculosis bacilli, and the alveolar macrophages, neutrophils, and dendritic cells (DCs) are activated and release numerous cytokines, chemokines, and antimicrobial peptides (19). Infected DCs migrate to the local draining lymph node under the influence of cytokines and chemokines to stimulate the differentiation of T helper 1 cells. The T helper 1 cells release cytokines (interferon gamma [IFN-γ] and tumor necrosis factor alpha [TNF-α]) at the site of infection and activate macrophages and DCs to produce cytokines and antimicrobial peptides for containment of the infection (20). Ultimately, a granuloma is formed, containing the bacilli in a latent state.

Hematogenous spread to other organ systems, including the CNS, may occur after one of two processes, as follows: (i), a short bacteremia may occur when M. tuberculosis is filtered into the local draining lymph nodes during primary TB infection, before granuloma formation; or (ii) the latent infection stage may progress to active TB disease due to a lapse or decrease in the immune response, especially in the elderly, immunocompromised, or very young individuals, thus leading to lung tissue destruction (19). M. tuberculosis bacilli bypass the alveolar epithelium through infected phagocytes or as free bacteria, and the latter has been linked to two bacterial proteins, namely, early secretory antigenic target 6 (ESAT-6) and culture filtrate protein 10 kDA (CFP-10), together with heparin-binding hemagglutinin adhesin (HBHA). TB bacilli may migrate across the blood-brain barrier (BBB) and blood-CSF barrier (BCSFB) through the following suggested mechanisms: (i) “Trojan horse,” in which M. tuberculosis bypasses the barriers via infected macrophages and neutrophils (19); or (ii) bacillary invasion of brain endothelium, mediated by M. tuberculosis pknD (*Rv0931c*) (21).

**FIG 1:**
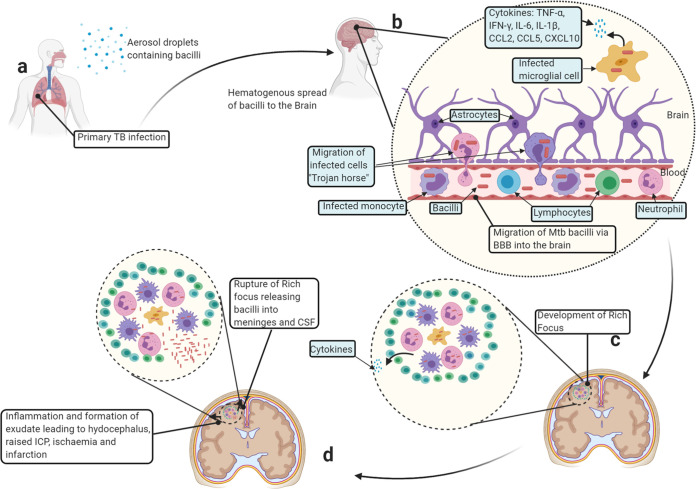
The generalized pathogenesis of tuberculous meningitis. (a) The host inhales aerosol droplets containing M. tuberculosis (Mtb) bacilli. Within the lungs, the bacilli may infect the alveolar macrophages, resulting in the formation of granuloma. The bacilli may then escape from a damaged granuloma or from the lungs during primary TB causing bacteremia, resulting in hematogenous spread of the bacteria into the brain. (b) Extracellular bacteria and infected cells may migrate through the blood-brain barrier (BBB) into the brain. Once in the brain, the bacilli infect microglial cells, which then together with infiltrating cells release cytokines and chemokines, leading to disruption of the BBB and influx of other uninfected immune cells into the brain. (c) This results in the formation of the granuloma “Rich focus.” (d) When the Rich focus ruptures, the bacteria are released into the subarachnoid space, leading to dissemination of the infection to the CSF and meninges. The release of bacteria into the meninges and CSF leads to meningeal inflammation and the formation of thick exudate. The thick exudate precipitates TBM signs.

In the brain, the TB bacilli initiate the development of tuberculous lesions (known as Rich foci) in the meninges or the subpial or subependymal surface (22). Rich and McCordock demonstrated through postmortem experiments that the rupture of these lesions releases M. tuberculosis into the subarachnoid space or ventricular system, causing granulomatous infection and subsequent inflammation of meninges (23). Recently, the role of miliary spread in addition to the generally accepted pathogenetic mechanism of the Rich focus has been revised, based on more recent clinical, postmortem, and epidemiological data (24). The onset of TBM takes less than 12 months from the time of primary infection in 75% of children (25). Poor outcomes in TBM are due to host inflammatory responses, which result in the formation of a thick exudate at the base of the brain. The dense basal exudate blocks the basal subarachnoid cisterns by the formation of adhesions, obstructing CSF flow and resulting in hydrocephalus and raised intracranial pressure. Further extension of the exudate results in (i) obliterative vasculitis of small proliferating blood vessels, leading to the development of focal and diffuse ischemic brain changes, whereas blockage of larger arteries results in infarction; and (ii) perineuritis, resulting in cranial nerve palsies; and in severe cases, (iii) direct parenchymal involvement (25, 26). A schematic representation of the route from inhalation of M. tuberculosis to the development of TBM is shown in Fig. 1.

### Clinical manifestation.

TBM typically presents as a subacute disease with many days or weeks (an average of 5 to 30 days) of nonspecific symptoms, including low-grade fever, malaise, headache, dizziness, vomiting, personality changes, and symptoms related to pulmonary TB (such as cough) (27, 28). Patients with advanced disease may present with more severe headache, altered mental status, stroke, hydrocephalus, and cranial neuropathies (27).

In children, clinical signs may include initial apathy or irritability that progresses to meningism, signs of raised intracranial pressure (such as abducens nerve palsy), and focal neurological signs (29). In adults, clinical signs may include neck stiffness, cranial nerve palsies (cranial nerve III, IV, VI, and VIII), confusion, and coma (29). Clinical motor deficits (monoplegia, hemiplegia, or paraplegia) occur in about 10% to 20% of cases (29, 30). Death is invariably inevitable if TBM is not treated.

### Immune response.

The host inflammatory response plays an important role in TBM pathology (31). Within the CNS, microglia are resident macrophages and are arguably the most prominent immune effector cells responsible for recognition and internalization of M. tuberculosis (32). Microglial cells and migrated infected neutrophils and macrophages become rapidly activated and can proliferate and increase the expression of different molecules and secrete cytokines and chemokines, which in turn modulate immune responses within the CNS (33). Such cytokines and chemokines released by infected microglial cells include TNF-α, IFN-γ, interleukin-6 (IL-6), IL-1β, CCL2, CCL5, and CXCL10 (34). The cytokines and chemokines may disrupt the BBB, allowing the influx of other uninfected cells (monocytes, neutrophils, and lymphocytes) (30). Infected cells (microglial cells and migrated cells) together with migrating uninfected cells lead to the formation of Rich focus.

Following the rupture of the Rich focus, release of M. tuberculosis into the subarachnoid space elicits a local T cell-mediated response characterized by caseating granulomatous inflammation (35). The role of T cells in TBM has been supported by several studies. The predominance of αβT cells and NK cells in the CSF of TBM patients was associated with better survival (36). Whole blood transcriptome analysis of children with TBM at different time points demonstrated that reduced T cell proliferation and immune responses is associated with disease progression (37). A recent study reported a similar reduction pattern; however, this study did not assess the changes over time (31).

Inflammatory mediators (cytokines and chemokines) including TNF-α, IFN-γ, IL-1β, IL-6, IL-8, and IL-10 are increased in the CSF of patients with TBM (17, 38–40). TNF-α has been linked to a protective role against M. tuberculosis, through the formation of granulomata. Studies on rabbit models of TBM have demonstrated that high levels of TNF-α in CSF were associated with worse outcomes (41). The use of TNF-α antagonists in combination with antibiotics improved survival and outcomes in rabbits (42). In contrast, CSF TNF-α levels of children treated for TBM did not show a significant decline over a 4-week period (43).

Other host mediators implicated in the pathology of TBM include matrix metalloproteinases (MMPs) and vascular endothelial growth factor (VEGF). Elevated levels of MMP-9, MMP-2, tissue inhibitor of metalloproteinase 1 (TIMP-1), and TIMP-2 were reported in CSF samples of pediatric patients with TBM (44). MMP-9 levels decreased significantly early in treatment; however, there was an increase during hospital stay, which was associated with better outcomes (44). MMP-2 and MMP-9 may be involved in the pathology of TBM due to their key role in the disruption of BBB and the BCSF barrier by breaking down the extracellular matrix that constitutes the barriers, which may in turn cause brain edema, tissue damage, and migration of blood-derived inflammatory cells (45). Therefore, the inhibition of MMPs, as proposed in one study, may be a potential strategy for management of complications seen in TBM (45). VEGF is a potent factor of vascular permeability and angiogenesis (46), is vasculotoxic, is prothrombotic, reduces cerebral blood flow, and produces nitric oxide as well as oxygen free radicals (47). In TBM, VEGF has been associated with brain edema and the disruption of BBB (48), with its induction being mediated by TNF-α (49).

In a recent study, transcriptional responses of pediatric TBM at a systemic level revealed upregulated innate cell populations, such as neutrophils, macrophages, resting dendritic cells, and plasma B cells, in the blood (31). Similarly, van Laarhoven et al. (36) observed increased numbers of mature neutrophils and classical monocytes in the blood of adult TBM patients versus healthy controls. Gene expression associated with CD4 and CD8 T cells was more predominant in healthy controls, while genes associated with T cell activation and their signaling were significantly downregulated in the TBM cases (31). Furthermore, TBM was also associated with proinflammatory inflammasome signaling pathways (31). This could suggest that innate and inflammasome responses play an important role in TBM and that reduced T cell response is associated with the disease. Although the current findings suggest a role for neutrophils and other innate cells in TBM immunopathogenesis, the roles of different immune cell subpopulations, including the mucosa-associated invariant T (MAIT) cells, a T cell subset that displays innate-like characteristics and which have only recently been described in the CSF of TBM patients (36), remain unclear. Further investigations are needed to assess the frequencies, characteristics, and responsiveness of immune cells in patients with TBM in order to understand the host responses underlying the disease pathology.

## CURRENT DIAGNOSTIC APPROACHES FOR TBM

### Clinical diagnostic criteria.

Owing to the inadequate performance of microbiological tests, there have been attempts to establish clinical diagnostic criteria for the diagnosis of TBM based on a combination of all the evidence from medical history, clinical assessment, and other relevant investigations (including CSF investigations and neuroimaging) (13). Despite numerous efforts to create clinical prediction rules to differentiate TBM from other meningitis based on these tests, standardized diagnostic criteria are still lacking. Thwaites et al. proposed a scoring system for the diagnosis of TBM in adults on the basis of clinical and basic laboratory findings (18). In 2010, Marais et al. developed TBM diagnostic criteria for clinical case definition in research, incorporating the findings of Thwaites et al., among others (17). This uniform research case definition classifies patients as “definite,” “probable,” “possible,” and “not TBM” and is based on a composite score of clinical findings, CSF findings, neuroimaging, evidence of TB elsewhere, and exclusion of alternative diagnosis (17). It is important to note that the uniform research case definition criteria were not designed for use in clinical practice. Hence, caution is needed when applying the research case definitions for TBM patient care (50).

### Microbiological diagnosis.

Smear microscopy is the most widely used rapid and inexpensive diagnostic test for TB; however, staining of CSF smears for acid-fast bacilli has poor sensitivity (about 10% to 15%) (51). Smear microscopy is therefore not reliable for the diagnosis of TBM. Mycobacterial culture, the gold standard for the diagnosis of TB, is recommended by the World Health Organization (WHO) for use in both adults and children, including for TBM. Although culture has a higher sensitivity (about 50% to 60%) for the diagnosis of TBM than that of other TB tests, its turnaround time (up to 8 weeks with solid media such as Lowenstein-Jensen [25, 51]) is a limitation. Although automated systems such as Bactec MGIT 960 have shown reduced average time to yield results (18 days versus 38 days), clinicians cannot afford to wait for culture results before treating patients, as death is a distinct possibility if empirical therapy is not initiated (30, 52). That notwithstanding, M. tuberculosis culture is still important for recovery of the bacilli needed for downstream phenotypic drug susceptibility testing (DST), as well as epidemiologic and sequencing-based studies.

### Molecular tests.

To overcome the limitations of the conventional laboratory diagnostic approaches, commercial nucleic acid amplification tests (NAATs) have emerged. These tests have the advantage of rapidity while simultaneously detecting drug resistance. In a recent systemic review and meta-analysis of 18 studies, NAATs were shown to provide better performance with pooled sensitivity and specificity of 96% and 92%, respectively (53). However, the diagnostic accuracy of NAATs is different depending on the specimen type, with respiratory specimens associated with better accuracy (53). These tests are therefore not reliable for ruling out TB from nonrespiratory specimens due to the lower sensitivity. For the diagnosis of TBM specifically, a systematic review and meta-analysis on NAATs reported a pooled sensitivity of 82% and specificity of 99% against culture (54) and sensitivity and specificity of 68% and 98%, respectively, against a composite reference standard (54). In line with these results, another meta-analysis reported a lower sensitivity of 64% but high specificity of 98% against CSF M. tuberculosis culture for commercial NAATs (55).

The Xpert MTB/RIF test (Cepheid, Sunnyvale, CA, USA), arguably the game changer, was developed for the rapid diagnosis of TB. It is an automated closed-cartridge system that allows the rapid (within 2 h) detection of both M. tuberculosis and rifampin (RIF) resistance simultaneously. The WHO recommends the GeneXpert test for the diagnosis of EPTB, including TBM with CSF specimen in both adults and children. The sensitivity of the GeneXpert test for TBM ranges from approximately 50% to 60% (56), with various performances reported. A study from Uganda reported that sensitivity improved from 28% to 72% when larger volumes (6 ml) of concentrated CSF were used, compared with 2 ml of uncentrifuged CSF (56). In another study, the GeneXpert test showed an overall sensitivity of 59.3% compared with clinical diagnosis (based on uniform case definition [17]) in adult TBM suspects (57), with another meta-analysis (1 retrospective study and 4 prospective studies) reporting pooled sensitivity and specificity values of 70% and 97%, respectively (55).

Xpert MTB/RIF Ultra (Xpert Ultra) was developed to overcome some of the shortcomings of the initial Xpert test, including the inadequate sensitivity. In a recent prospective cohort study, Xpert Ultra demonstrated a sensitivity of 70% for probable or definite TBM (diagnosed based on uniform case definition [17]) compared with 43% obtained by either Xpert or culture (58). Compared with either Xpert or culture, Xpert Ultra diagnosed TBM in HIV-infected adults with a sensitivity of 95% (21/22 cases) (58). Although Ultra demonstrated improved performance, it does not appear to be adequate to rule out TBM due to concerns over low negative predictive value (NPV).

Another promising NAAT which will potentially be suitable for use in resource-limited settings, as it does not require expensive instruments or expertise and yields results in 60 minutes, is the loop-mediated isothermal amplification (LAMP) test (59). When investigated as a diagnostic test for TBM, LAMP showed potential with sensitivity between 88% and 96% and specificity of 80% to 100% (59, 60). Another NAAT that is commercially available (the Amplicor TB PCR test) has a sensitivity of ∼40% and specificity of ∼90% to 100% in the diagnosis of TBM, as reported in a recent review (14). The requirements of trained laboratory staff and high costs limit the wide use of the test (14). The Gen-probe amplified M. tuberculosis direct test (MTD), another NAAT, was initially intended for the detection of M. tuberculosis in respiratory specimens. When CSF samples were used in the test, it showed potential in the diagnosis of TBM, with pooled sensitivity of 86% and specificity of 99% (54). Another commercial NAAT, the Genotype MTBDRplus is a molecular line probe assay that targets specific genes for M. tuberculosis complex detection, as well as rifampin (RIF) and isoniazid (INH) susceptibility (61). When evaluated in a few TBM cases, the sensitivity of the test was 33%, with specificity of 98% against a clinical reference standard (61). While most NAATs have shown potential as diagnostic tests for TBM, more data on the performance of the tests are still required. Overall, commercial NAATs have demonstrated high specificity but, generally, suboptimal sensitivity for TBM, whereas Xpert Ultra, although promising, can still miss up to 30% of TBM patients. Despite improved diagnostic performance, both the GeneXpert and Xpert Ultra tests cannot rule out TBM due to their low negative predictive value (51, 62). Although NAATs are a major diagnostic advance, they are still inadequate to replace culture methods. The CRISPR-M. tuberculosis and metagenomic next-generation sequencing (mNGS) technologies (reviewed in reference 63), may improve the detection of M. tuberculosis in CSF samples with low bacillary load. However, further investigations are required to ascertain the performance of these methods.

### Brain imaging.

Brain imaging techniques, such as computed tomography (CT) and magnetic resonance imaging (MRI), are part of the clinical diagnostic assessment of TBM. Contrast-enhanced CT imaging reveals basal meningeal exudates specific for TBM and predicts poor outcomes (30). However, neurological signs or features (such as infarcts and hydrocephalus) revealed by CT imaging lack diagnostic specificity for TBM, mainly because similar features are seen in other infectious and noninfectious diseases (30). MRI has been found to have superior diagnostic abilities compared with CT (64). This includes better detection of basal meningeal enhancement and infarcts (especially in the brain stem) and early infection (64). The main common limitation to these brain imaging techniques is that CT scans are normal in about 30% of individuals at an early stage of TBM, while MRI scans are normal in about 15% (30). Furthermore, both CT and MRI evaluations are usually carried by medical experts in a tertiary care setting and are mostly not available in primary care settings or resource-limited settings (MRI more so than CT) (65).

### Immune response-based diagnosis.

Immunodiagnostic tests, such as interferon gamma release assays (IGRAs), are primarily used for the diagnosis of M. tuberculosis infection. IGRAs measure the IFN-γ produced by lymphocytes when stimulated with M. tuberculosis-specific antigens. As these tests cannot differentiate latent TB infection (LTBI) from active TB disease, their use in the diagnosis of active TB is discouraged (66). The WHO recommends the use of IGRAs for LTBI testing in individuals who are at risk, including people living with HIV and infants and children aged 5 years and younger who are household contacts of pulmonary TB patients in both low- and high-TB burden settings (66). In TBM, a moderate diagnostic accuracy was reported for CSF IGRA, with sensitivity of 77% and specificity of 88% (67). The most commonly used immunodiagnostic modality in TBM management is the measurement of adenosine deaminase (ADA). ADA is an enzyme that is released by lymphocytes and plays an important role in the proliferation and differentiation of T cells. As the release of ADA from T cells has been associated with cell-mediated immune responses to tubercle bacilli, the measurement of levels of the enzyme in CSF is being done as an approach for the diagnosis of TBM (68–70). A meta-analysis of 20 studies on the accuracy of ADA reported a pooled sensitivity of 89% and specificity of 91% in the diagnosis of TBM (71). However, evidence about the clinical usefulness of CSF ADA is contradictory. Ekermans and colleagues reported that at an optimal cutoff of 2.0 U/liter, the sensitivity and specificity of CSF ADA was 85.9% and 77.7%, respectively (72). The study further showed that an optimal cutoff value for the routine diagnosis of TBM could not be established, as many cases were missed (72). Furthermore, high numbers of false positives and limited utility were reported for CSF ADA in another study on HIV-infected individuals (73). The main concern with the use of ADA in practice is the fact that similar levels of the protein have been documented in CSF from patients with other infective pathologies, including bacterial meningitis and ventriculitis, thereby making interpretation of the results difficult. ADA is therefore not useful in settings where the differential diagnosis is broad (72). As CSF ADA results may be misleading, clinicians should be aware of its limitations when making TBM diagnostic decisions. The poor standardization of ADA assays and the fact that ADA results are dependent on the integrity of the specimen (72) are further concerns. In summary, none of the currently available methods is adequate for use as a stand-alone test for the diagnosis of TBM. New and improved diagnostic methods are therefore urgently needed.

## POTENTIAL OF NOVEL BIOMARKER-BASED APPROACHES

In the search of better TB diagnostic tools, recent studies have investigated several alternative approaches, including the measurement of protein concentrations in biological fluids, transcriptional molecules, and metabolites as biomarkers for TB. Several attempts are being made at detecting such biomarkers in easily obtainable specimens, such as blood, urine, and saliva, among others, with the need for such nonsputum-based approaches being deemed a high priority by the WHO (74).

### Host protein biomarkers.

Several studies have proven that the measurement of inflammatory proteins, such as cytokines, chemokines, acute phase proteins, and growth factors, can differentiate TB from other infections (reviewed in reference 75). Earlier studies evaluated the value of alternative proteins other than IFN-γ that were detected in supernatants following the stimulation of blood cells with M. tuberculosis-specific antigens using multiplex immunoassays, mainly the Luminex platform (76–78). As such studies were based on overnight stimulation assays, most of the recent studies have focused on the evaluation of host markers in unstimulated specimens, including serum (79), plasma (80), urine (81), and saliva (82), given that such biomarkers may be more easily translated into point-of-care tests.

In a study conducted in China on adult patients with CNS infection, including TBM (*n* = 17), purulent meningitis (*n* = 13), and cryptococcal meningitis (*n* = 13), CSF levels of IL-1β, TNF-α, IFN-γ, IL-6, IL-4, IL-10, IL-17A, IL-17F, and CD40L were ≥2-fold higher in the TBM group than in the control group (83), with IL-6 reported as the most important cytokine for differentiating CNS infection from controls (83). CSF glucose and the CSF/blood glucose ratio were negatively correlated with CSF IL-6 levels in patients with CNS infection, thus revealing the potential of combining CSF IL-6 and CSF glucose as a biomarker for CNS infection (83). In another Chinese study that included patients with viral meningitis, encephalitis, and bacterial meningitis and patients with intracranial metastatic tumor as controls, CSF Delta-like 1 ligand (DLL) levels showed promise in diagnosing TBM with a sensitivity of 87.1%, specificity of 99.1%, negative predictive value (NPV) of 92.2%, and positive predictive value (PPV) of 98.2%, at a cutoff value of >1.0 ng/ml (84). Similarly, serum DLL levels were also higher in the TBM group and diagnosed TBM (cutoff value of >6.0 ng/ml) with sensitivity of 82.3%, specificity of 91.0%, PPV of 83.6%, and NPV of 90.2% (84). In contrast, a Ugandan study conducted in HIV-infected patients reported poor sensitivity (32%) but high specificity (98%) (cutoff value of 1,150 pg/ml) for DLL1 in the diagnosis of TBM (85). Another protein (high mobility box-1; HMGB1), a damage-associated molecular pattern (DAMP) protein that plays a role in inflammation, was also shown to have potential in the diagnosis of TBM (sensitivity and specificity of 61.02% and 89.94%), respectively, at a (cutoff value of 3.4 ng/ml) in another study (86). Other studies that evaluated the value of various protein biomarkers as TBM diagnostic candidates include a South African study by Visser et al. (87), which identified a three-marker CSF biosignature of IL-13, VEGF, and cathelicidin LL-37, that showed potential (sensitivity of 52.0%), specificity of 95.0%, PPV of 91.0%, and NPV of 66.0% in the diagnosis of TBM in young children (87). When assessed in a more recent study, this three-marker biosignature diagnosed TBM with improved sensitivity of 95.7% at the cost of specificity (37.5%), with better results obtained (sensitivity of 91.3% and specificity of 100%) when IL-13 and LL-37 were replaced by IFN-γ and myeloperoxidase (MPO), which was also for the diagnosis of TBM in children (88). Multiple new biomarkers, including a new four-marker biosignature (soluble intracellular adhesion molecule 1 [sICAM-1], MPO, IL-8, and IFN-γ) and various individual biomarkers, including IFN-γ, MIP-4, CXCL9, CCL1, RANTES, IL-6, TNF-α, MPO, MMP-9, MMP-8, complement component 2 (CC2), IL-10, PAI-1, CXCL8, IL-1b, A1AT, CXCL10, granulocyte colony-stimulating factor (G-CSF), CC4, CC4b, granulocyte-macrophage-CSF (GM-CSF), platelet-derived growth factor (PDGF) AB/BB, apolipoprotein (Apo)-AI, metallo-β-lactamase (MBL), ferritin, CC5a, SAP, and CC5, were shown to have potential for childhood TBM diagnosis in the same study (88).

Despite the promise shown by the CSF host inflammatory biomarker-based studies described above, the procedure for the collection of CSF (lumbar puncture) may be a limitation in the implementation of CSF-based tests in resource-limited settings. Blood-based biomarkers, which have shown potential and are being developed into point-of-care tests for the diagnosis of pulmonary TB (79, 89), may be alternatives in TBM diagnosis. When evaluated as a tool for the diagnosis of TBM in children, a modified version of an adult serum seven-marker signature (C-reactive protein [CRP], IFN-γ, IP-10, CFH, Apo-A1, SAA, and NCAM) diagnosed TBM with moderate accuracy (sensitivity of 73.9% and specificity of 66.7%) (90). In the same South African study, a new three-marker serum biosignature (adipsin, Aβ42, and IL-10), which diagnosed childhood TBM with sensitivity of 82.6% and specificity of 75.0%, alongside several individual candidate biomarkers were identified (90). Given the potential shown in these studies, there is a need for further discovery and validation of similar biomarkers, followed by incorporation of the most promising candidates into point-of-care tests. The added benefit of blood-based biomarkers is the possibility of detecting them in fingerprick blood, as is currently being done for new pulmonary TB-based prototype tests (https://www.triagetb.com/).

### Host transcriptional biomarker-based signatures.

Transcriptomics has become a popular approach for biomarker discovery, with several infectious diseases, including TB biosignatures, being discovered using techniques such as RNA sequencing, quantitative real-time PCR, and microarrays. It is suggested that quantifying the shifts in RNA abundances triggered by diseases could help in identifying diagnostic, disease-associated, and treatment response biomarkers. Recent studies have identified gene signatures that predict the onset of active TB several months before the onset of symptoms (91), signatures for the prediction of progression from latent TB infection to active TB in household contacts (92), diagnosis of TB (93, 94), and monitoring of TB treatment response (95). Most of these investigations have been adult, pulmonary TB-based studies, with one study reporting on the up-, or downregulation of 796 genes (398 and 398, respectively) in brain tissues of TBM patients who were coinfected with HIV (96). Of importance, four gene products, namely, glial fibrillary acidic protein (GFAP), serpin peptidase inhibitor clade A member 3 (SERPINA3), thymidine phosphorylase (TYMP/ECGF1), and heat shock 70 kDA protein 8 (HSPA8), were confirmed to be abundant in TBM patients with HIV coinfection (96). As this study compared TBM patients with individuals who succumbed to road traffic accidents, the utility of these genes as candidate TBM diagnostic biomarkers is unknown. In addition to evaluating the usefulness of these genes as TBM diagnostic candidates, further work is required for the identification and validation of TBM-specific biosignatures in well-designed TBM diagnostic studies. Given that prototype fingerprick blood-based mRNA signature tests currently exist (97), validated TBM transcriptomic biosignatures could be further incorporated into such platforms, followed by large-scale field trials against acceptable reference standards.

### Host miRNA biosignatures.

MicroRNAs (miRNAs) are a class of conserved noncoding small RNAs (21 to 25 nucleotides long), which play an important role in the regulation of gene expression and other biological processes, including cell proliferation, cell differentiation, organ development, apoptosis, immune response, angiogenesis, and onset of disease (98, 99). Altered expression of miRNAs has been associated with TB (100). In a study including 112 children with TBM and 130 healthy controls, miR-29a expression in peripheral blood mononuclear cells (PBMCs) showed potential in the diagnosis of TBM, with a sensitivity of 67.2% and specificity of 88.5 and a sensitivity of 81.1% and specificity of 90% when evaluated in CSF (101). When used in combination, CSF plus PBMC miR-29a expression diagnosed pediatric TBM with sensitivity of 84.4% and specificity of 95.4% (101).

In a recent genome-wide miRNA analysis study performed on adult PBMCs and CSF samples (99), a combination of four miRNAs (miR-126-3p, miR-130a-3p, miR-151a-3p, and miR-199a-5p) discriminated TBM from viral meningitis (VM) in PBMCs with sensitivity of 90.6% and specificity of 86.7% and discriminated TBM from healthy controls with sensitivity of 93.5% and specificity of 70.6% (99). Three CSF-based miRNAs (miR-126-3p, miR-130a-3p, and miR-151a-3p) also showed potential in discriminating between TBM and VM (99), with miR-199a-5p levels undetectable in CSF (99). The four-marker PBMC miRNA signature (miR-126-3p, miR-130a-3p, miR-151a-3p, and miR-199a-5p) was validated in an independent sample set in the same study with sensitivity of 81.8% (9/11) and specificity of 90.0% (9/10) in distinguishing TB and VM and sensitivity of 81.8% (9/11) and a specificity of 84.6% (11/13) in discriminating TBM from other non-TBM patients (99). Three exosomal miRNAs (miR-20b, miR-191, and miR486) also showed potential as biomarkers for discriminating TBM from non-TBM disease when used in combination with electronic health records (EHRs) in another study, diagnosing TBM with sensitivity of 94% and specificity of 95% (102). Taken together, these studies show that miRNA-based biosignatures have potential as candidate TBM diagnostic biomarkers. However, more studies on their potential value are required, including studies done at multiple field sites in both adults and children.

### Metabolic biosignatures.

Metabolomics is an emerging powerful and advanced omics platform, which may be useful in the identification of novel diagnostic biomarkers. The technique is used to identify metabolites that are associated with certain physiological or pathological conditions. Several studies have demonstrated significant differences in amino acids and energy metabolism in CSF samples of TBM patients compared with other groups, including patients with viral, bacterial, and cryptococcal meningitis (103–106). However, the diagnostic accuracy of the metabolites identified was not reported. In a study that investigated urine metabolic biomarkers in 12 children with TBM and 29 controls, a host biosignature (*SUM-4*) generated from the sum of urinary concentrations of methylcitric acid, 2-ketoglutaric acid, quinolinic acid, and 4-hydroxyhippuric acid, separated TBM from other groups with an area under the receiver operator characteristic (ROC) curve (AUC) of 96.6% (107). These largely small, proof-of-concept studies provide evidence that host metabolomic biomarkers may be useful in the diagnosis of TBM. However, further work is required in this field, coupled with work focusing on the development of end user-friendly detection devices for the measurement of any candidate metabolites, preferably at the point of care.

## CONCLUSIONS

The diagnosis of TBM remains challenging, mainly due to difficulties in the direct detection of M. tuberculosis bacilli in CSF and other specimens from patients who are suspected of having the disease. The recently introduced Xpert MTB/RIF Ultra has shown promise in detecting paucibacillary TB, including diagnosing more TBM cases than the previous version. However, it may still fail to rule out TBM due to inadequate negative predictive value. Furthermore, its implementation in resource-limited settings will be hampered by the same issues that hindered the successful roll out of the GeneXpert MTB/RIF test in such settings (108, 109). None of the currently available diagnostic tools is adequate as a stand-alone method for the definite diagnosis of TBM. Therefore, CSF microscopy, mycobacterial culture, and molecular tests, such as GeneXpert, Xpert Ultra, and other NAATs, should all be performed for the diagnosis of TBM, in settings where this is possible. The TB field has recently seen much activity in the discovery, validation, and development of novel biomarker-based tests, but most of this activity is for the management of pulmonary TB, especially in adults. The few host biomarker-based projects that have focused on TBM have shown that the targets described in the WHO target-product profiles (TPPs) for a nonsputum biomarker test for the diagnosis of extrapulmonary TB (sensitivity of at least 80% in CSF samples for microbiologically confirmed TB and specificity of 98%, or as specific as the Xpert MTB/RIF) (74) may be achievable or approachable given the recent promising findings (88, 90, 99, 101, 102). However, much work is still required in the refinement and validation of the proposed biomarkers. Furthermore, such biomarker-based approaches will make the most impact only if further developed into easy-to-use diagnostic tests, especially tools that are implementable at the point of care, in resource-limited settings.

## DIRECTIONS FOR FUTURE RESEARCH

- Traditionally, TB diagnostic tests have been developed and largely validated in adults with pulmonary TB. Research focusing on the development and validation of tools in children and especially tests that may be useful in the diagnosis of EPTB, including TBM, should be encouraged.
- Assessment of biomarkers produced after stimulation of blood cells with M. tuberculosis antigens (110, 111) has been shown to possess diagnostic potential in other extrapulmonary forms of TB (112–114). Such approaches should be explored in other EPTB types, including TBM.
- Approaches that have resulted in potentially useful pulmonary TB signatures in noninvasive and easily obtainable specimens, including saliva (82, 115, 116) and urine (81, 117), require evaluation in TBM and should be encouraged.
- Further work needs to be done to validate the different inflammatory host biomarker signatures reviewed in the current study (Table 1). Such future work should focus on the following:
  - Evaluation of the signatures in large independent cohorts of both adults and children that are recruited after clinical suspicion of having meningitis, prior to the confirmation of TBM or no TBM.
  - Evaluation of the influence of HIV infection and other comorbidities on biomarker accuracy.
  - Inclusion of participants from different geographical areas in the evaluation and validation of biomarkers, such as to assess global applicability.
  - Incorporation of the most promising globally relevant biosignatures into point-of-care tests, followed by field trials of the tests in multiple settings.
  - While validation of the few transcriptomic, metabolomic, and miRNA candidate biomarkers that have so far been identified (Table 1) is ongoing, further work, including new biomarker discovery in new, well-designed TBM studies in which controls are individuals suspected of having TBM as would be obtained in routine clinical practice, is encouraged.
  - If the goal of developing a useful TBM biomarker-based point-of-care test remains elusive, the inclusion of different biomarker-based tests in a uniform research omics-based case definition may be beneficial. A uniform research omics-based case definition could be based on different blood and CSF validated biomarker signatures/omics-based tests, which may diagnose TBM with optimal accuracy when combined.
  - To further enhance our knowledge of the immunology and pathogenesis of TBM (Fig. 1), further investigations of the immune cell populations, characteristics, and responses at both the site of disease (CSF) and peripheral (blood) in TBM patients and appropriate controls should be encouraged. Such knowledge may shed light on new potential vaccine and host-directed therapeutic targets.
  - Following the development of new tools for TBM, evaluation of the accuracy of the tools against appropriate benchmarks, e.g., the TPPs proposed by the WHO, is encouraged (74).

**TABLE 1:** Host biomarkers with potential for use in diagnosis of tuberculous meningitis

Category	Biomarker	Sample	Sample size of:			Location	Approach	Sensitivity (%)	Specificity (%)	Intended application^*a*^	Reference
			TBM cases	Controls	Total						
Host protein markers	Delta-like 1 ligand	CSF^*b*^	62	111	173	China	ELISA^*c*^	87.1	99.1	TBM vs VM, BM, nondiagnosed group	Peng et al. (84)
	Delta-like 1 ligand^*d*^	CSF	62	111	173	China	ELISA	82.3	91.0	TBM vs VM, BM, nondiagnosed group	Peng et al. (84)
	HMGB1	CSF	59	169	228	China	ELISA	61.02	89.94	TBM vs control patients	Chen et al. (86)
	3-host marker signature (VEGF + IL-13 + cathelicidin LL-37)	CSF	56	55	111	South Africa	Multiplex cytokine assay, ELISA (LL-37)	52.0	95.0	TBM vs Non-TBM	Visser et al. (87)
	3-host marker signature (VEGF + IL-13 + cathelicidin LL-37)	CSF	23	24	47	South Africa	Multiplex cytokine assay, ELISA (LL-37)	95.7	37.5	TBM vs no TBM	Manyelo et al. (88)
	3-host marker signature (VEGF + IFN-γ + MPO)	CSF	23	24	47	South Africa	Multiplex cytokine assay	91.3	100.0	TBM vs no TBM	Manyelo et al. (88)
	4-host marker signature (sICAM + MPO + IL-8 + IFN-γ)	CSF	23	24	47	South Africa	Multiplex cytokine assay	96.0	96.0	TBM vs no TBM	Manyelo et al. (88)
	7-host marker signature (CRP + IFN-γ + IP-10 + CFH + Apo-A1 + SAA + NCAM1)	Blood	23	24	47	South Africa	Multiplex cytokine assay	73.9	66.7	TBM vs no TBM	Manyelo et al. (90)
	3-host marker signature (adipsin + Aβ42 + IL-10)	Blood	23	24	47	South Africa	Multiplex cytokine assay	82.6	75.0	TBM vs no TBM	Manyelo et al. (90)
Host RNA	792 up- or downregulated genes (of importance: GFAP, SERPINA3, TYMP/ECGF1, and HSPA8)	Brain tissues	5	4	9	India	Microarray and immunohistochemistry validation	NR^*e*^	NR	TBM vs individuals who succumbed to road traffic accidents	Kumar et al. (96)
Host microRNA	Mir-29a	PBMCs	122	130	252	China	qRT-PCR^*f*^	67.2	88.5	TBM vs HC	Pan et al. (101)
		CSF	122	130	252	China	qRT-PCR	81.1	90.0	TBM vs HC	Pan et al. (101)
	4-host miRNA marker signature (miR-126-3p + miR-130a-3p + miR-151a-3p + miR-199a-5p)	PBMCs	32	64 (30 VM, 34 HC)	96	China	Genome-wide microarray, qPCR independent validation	90.6	86.7	TBM vs VM	Pan et al. (99)
								93.5	70.6	TBM vs HC	
Metabolic markers	16 NMR^*g*^ metabolites	CSF	33	73 (30 nonmeningitis controls from South Africa and 43 neurological controls from the Netherlands)	106	South Africa	Untargeted magnetic resonance (^1^H NMR)-based metabolomics analysis	NR	NR	TBM vs controls	Mason et al. (103)
	Alanine, glycine, lysine, proline, and asparagine	CSF	33	34	67	South Africa	GC-MS^*h*^	NR	NR	TBM vs controls (suspected meningitis)	Mason et al. (104)
	25 key metabolites	CSF	18	20	38	China	^1^H NMR-based metabolomics	NR	NR	TBM vs VM	Li et al. (105)

a TBM, tuberculous meningitis; VM, viral meningitis; BM, bacterial meningitis; HC, healthy controls.

b CSF, cerebrospinal fluid.

c ELISA, enzyme-linked immunosorbent assay.

d At a different cutoff value.

e NR, not reported.

f qRT-PCR, quantitative real-time PCR.

g NMR, nuclear magnetic resonance.

h GC-MS, gas chromatography-mass spectrometry.
